# A New Mouse Avatar Model of Non-Small Cell Lung Cancer

**DOI:** 10.3389/fonc.2015.00052

**Published:** 2015-03-03

**Authors:** Maria Veronica Russo, Alice Faversani, Stefano Gatti, Dario Ricca, Alessandro Del Gobbo, Stefano Ferrero, Alessandro Palleschi, Valentina Vaira, Silvano Bosari

**Affiliations:** ^1^Division of Pathology, Fondazione IRCCS Ca’ Granda Ospedale Maggiore Policlinico, Milan, Italy; ^2^Department of Pathophysiology and Transplantation, Doctorate School in Molecular and Translational Medicine, University of Milan, Milan, Italy; ^3^Center for Preclinical Surgical Research, Fondazione IRCCS Ca’ Granda Ospedale Maggiore Policlinico, Milan, Italy; ^4^Department of Medical Biotechnology and Translational Medicine, University of Milan, Milan, Italy; ^5^Department of Biomedical, Surgical and Dental Sciences, University of Milan, Milan, Italy; ^6^Division of Thoracic Surgery and Lung Transplantation, Fondazione IRCCS Ca’ Granda Ospedale Maggiore Policlinico, Milan, Italy; ^7^Istituto Nazionale Genetica Molecolare “Romeo ed Enrica Invernizzi” (INGM), Milan, Italy

**Keywords:** NSCLC, patients-derived tumor xenografts, tissue slices, organotypic culture, miRNA

## Abstract

**Introduction:** Lung cancer remains the leading cause of tumor-related deaths, despite advances in the understanding of the disease pathogenesis and in its clinical treatment. It is crucial to develop novel technologies to discover disease biomarkers and predict individual therapy response.

**Materials and methods:** We established 48 patients-derived tumor xenografts (PDTXs) implanted in the subrenal capsule of immunodeficient mice using thin, precision-cut tumor tissue slices, derived from five patients affected by non-small cell lung cancer. Twenty-six tissue slices were immediately processed and implanted at sample recovery [patients-derived tumor xenografts derived from fresh tissue (dPDTX)], whereas the remaining sections were cultured on specific organotypic supports at 37°C and 5% CO_2_ for 24 h before grafting [patients-derived tumor xenografts derived from cultured tissue (cPDTX)]. At sacrifice, xenografts tissue morphology, proliferation (Ki67), and histotype markers were analyzed. Oncogenic miRNAs profiles were assessed in PDTXs, human tumors, and serum from one patient.

**Results:** Xenografts retained the original cancer features and there were no differences between dPDTXs and cPDTXs. Squamous cell carcinoma (SCC) xenografts showed a higher engraftment rate than adenocarcinoma (AC)-derived tumors. At basal time, Ki67 levels were higher in SCCs than in ACs, and the expression levels of genes associated to a stem cell-like phenotype were also more expressed in SCC samples. The analysis of oncogenic miRNAs showed that circulating miR-19b, -21, and -210 levels were correlated with higher Ki67 expression in xenografts.

**Conclusion:** Our study implemented the PDTX model with thin, precision-cut tumor slices from small tumors, which could be useful for clinical applications and predictive purposes. The different engraftment success is likely determined by tumor histotype, high proliferation index, and the expression of genes essential for cancer stem cells maintenance. Our PDTXs model could be a valid tool to expand primary tumors for the discovery of new biomarkers and explore therapeutic options.

## Introduction

Lung cancer is the principal cause of cancer-related deaths worldwide. Non-small cell lung cancer (NSCLC) is the major form, accounting for about 85% of all lung tumors, with three histological subtypes: adenocarcinoma (AC), squamous cell carcinoma (SCC), and large cell carcinoma (1).

Although much effort is focused in early disease detection, NSCLC is often diagnosed at advanced stages, not suitable for surgical resection and characterized by a dismal prognosis. Moreover, despite several advances in our insights of molecular mechanisms of lung tumorigenesis and therapeutic response, the current therapies offer only modest survival benefits and drug resistance commonly arise (2). This failure may at least in part be due to the pathological and molecular heterogeneity characteristic of this disease (3).

It is crucial to develop novel technologies in order to discover new biomarkers and predict individual therapy response. *In vivo* models able to mimic patients’ malignancies are useful tools to target this need.

The patients-derived tumor xenografts (PDTXs), also called mice avatar, are *in vivo* models which are based on the graft of human tumor fragments in immunocompromised mice (4). Xenografts retain the histopathological and genetic features of the original cancers making the PDTX a valid method to expand primary tumors, predict cancer response to therapy, and to determine new therapeutic markers (5). However, different factors including tumor histotype, grade, and the site of the transplant can influence the success of the graft. Moreover, tissue fragments must be rapidly processed and the original material is often insufficient (6, 7).

Recently, we perfected an *ex vivo* platform suitable to culture thin, precise-cut tissue slices from a variety of pathological and normal tissues in a biological context that strictly resemble the original microenvironment (8, 9).

In order to overcome PDTXs limits, we combined PDTX method with our *ex vivo* tissue culture platform. We established 48 subrenal capsule grafts using thin, precise-cut, 300 μm-thick tumor tissue slices derived from five NSCLC patients. Tissue slices were immediately processed and implanted at the sample recovery [patients-derived tumor xenografts derived from fresh tissue (dPDTX)] or cultured for 24 h and subsequently grafted with the same technique [patients-derived tumor xenografts derived from cultured tissue (cPDTX)].

## Materials and Methods

### Lung cancer specimens

Primary human lung tumors were obtained from five patients who underwent surgery for therapeutic purposes at Fondazione IRCCS Ca’ Granda, Policlinico Hospital (Milan, Italy). The patients’ clinicopathologic features are shown in Table 1. Patients did not receive neo-adjuvant chemotherapy and/or radiotherapy before surgery. Blood samples were collected from two patients. Informed consent was obtained from all patients and the study was approved by the Institutional Review Board of the Fondazione IRCCS Ca’ Granda.

**Table 1:** **Clinicopathological characteristics of NSCLC studied (*n* = 5)**.

Sample	Gender	Age	Histotype	G	T	N
SCC1	M	45	Squamous cell carcinoma	G2	pT2a	N1
AC2	F	83	Adenocarcinoma	G3	pT1b	N2
AC3	F	75	Adenocarcinoma	G3	pT1a	N0
SCC4	M	53	Squamous cell carcinoma	G3	pT2a	N0
AC5	M	74	Adenocarcinoma	G2	pT2a	N0

*Tumor stage according to TNM staging systems and tumor grade are indicated*.

### Tissue slices preparation

Lung cancer tissue slices (300 μm-thick) were obtained through serial cutting of the individual samples using a vibratome VT1200 (Leica Microsystems, Wetzlar, Germany), as previously described (8). Tissue processing was performed within 20 min after surgical resection. For all specimens, the first tissue slice was collected (baseline sample, *T*_0_), formalin-fixed and paraffin-embedded (FFPE) for morphological and immunohistochemical analyses.

### Organotypic tissue cultures

Tissue slices were cultured as already described (8). Briefly, NSCLC sections were cultured on specific organotypic inserts (Millipore, Darmstadt, Germany) for 24 h at 37°C in a humidified incubator with 5% CO_2_. Culturing media consisted of Ham F-12 media supplemented with 20% inactivated FBS (Life Technologies, Carlsbad, CA, USA), 100 U/mL penicillin (Life Technologies), 100 μg/mL streptomycin (Life Technologies), 2.5 μg/mL amphotericin B, and 100 μg/mL of kanamycin (Sigma Aldrich, St. Louis, MO, USA). For each tumor, a tissue slice was harvested after 24 h of culture, FFPE, and used as control [24 h culture control (NTC24)] for morphological and immunohistochemical analyses.

### Xenografts establishment

Three hundred micrometer-thick tissue slices were cut into multiple 3 mm × 3 mm pieces and submerged in sterile PBS supplemented with antibiotics (10% Pen Strep; Life Technologies). Forty-eight tissue fragments were grafted under the renal capsule of 6–8 weeks old CD1 athymic mice (Charles River Laboratories, Calco, Italy; one graft/kidney) within 1 h, as previously described (10). Twenty-six tissue slices were immediately processed and implanted at sample recovery (dPDTX) whereas the others were cultured for 24 h and subsequently grafted (cPDTX). At 1.5, 3, and 6 months from the graft, mice were sacrificed and xenografts were collected. Individual tumor volumes were calculated as *V* = (*LWS*)/2 where *L* and *W* are longer and shorter axis, respectively, and *S* is the xenograft thickness. *L* and *W* were measured using a caliper, whereas *S* was determined by DMD108 microscope (Leica Microsystems). The variation of tumor size was expressed as the final volume (*V*_f_) and initial volume (*V*_i_ = 1.35 mm^3^) ratio. Tumors were considered engrafted when they retained viable cancer tissue (engraftment rate). Lungs, liver, the contralateral normal kidney, and spleen were harvested and FFPE for evaluation of metastatic foci. For 42 mice, blood sample was collected at sacrifice by cardiac puncture.

Animal care and experiments were performed in accordance with the Principles of Laboratory Animal Care (NIH Publication No. 86-23, revised 1985) and approved by the Local Committee for Experimental Animal Research. Mice were anesthetized by i.p. injection with fresh 2,2,2-tribromoethanol (Avertin, Sigma Aldrich) and sterile practices were followed during the surgical procedure. SCC4 mice were sacrificed after heavy sedation with a dose of 500 mg/kg of avertin by i.p (Sigma Aldrich) at 1.5 and 3 months because they showed pain symptoms with a progressive degeneration of their physical condition starting from the first month after grafting.

### Morphological and immunohistochemical analyses

In order to assess tissue morphology, hematoxylin and eosin (H&E) staining was performed. Tumor tissues immunoreactivity for Ki67, CK5/6, P63, TTF1, NAP-A, CD31, vimentin (antigen retrieval by EDTA at 95°C for 36 min), or SOX2 (antigen retrieval by citrate buffer at 95°C for 44 min) was analyzed. All primary antibodies except SOX2 (Cell Signaling, Danvers, MA, USA) were from Ventana Medical Systems (Roche Group, Tucson, AZ, USA). Individual primary antibodies were detected using specific secondary antibodies and visualized by 3,3′-diaminobenzidine (DAB) and counterstained with hematoxylin. Negative controls were prepared in the absence of primary antibody and included in each reaction. Three investigators (Silvano Bosari, Alessandro Del Gobbo, and Stefano Ferrero) independently examined and scored the slides. When differences arose, the cases were reviewed until a consensus was reached. For quantification of proliferative activity, a Ki67 score was determined as the percentage of positive tumor cells. Immunoreactivity for CK5/6, P63, TTF1, NAP-A was quantified as the percentage of positive tumor cells. SOX2 levels were determined using a two-score system for percentage of positive cells and intensity of staining. The staining intensity was expressed in a scale of 0 (absent staining) to 3 (strong staining). Representative images were acquired using a DMD108 system (Leica Microsystems) and contrast/brightness was adjusted using identical settings with Photoshop (Adobe Systems Inc., San Jose, CA, USA).

### RNA purification and reverse transcription

Total RNA was purified from FFPE samples of NSCLC tissues and corresponding non-neoplastic lung parenchyma using the MasterPure RNA purification kit (Epicentre, Madison, WI, USA) following the manufacturer’s instructions. RNA from serological specimens of human or murine origin was isolated using miRNeasy serum/plasma kit (Qiagen, Limburg, Netherlands) according to the supplier’s protocol. Before RNA purification, an exogenous small RNA (cel-miR-39; Qiagen) was added to each serum samples for normalization of extraction procedures. RNA was quantified spectrophotometrically. For gene expression quantification, 300 ng of total RNA were reverse-transcribed using the high-capacity cDNA reverse transcription kit (Life Technologies) in a *V*_f_ of 20 μL.

Conversely, for miRNAs profiling, 33 ng of total RNA were reverse-transcribed using the TaqMan MicroRNA reverse transcription kit with the Megaplex RT Primers Human Pool A v.2.1 and then preamplified using the TaqMan PreAmp Master Mix with the Megaplex PreAmp Primers Human Pool A v.2.1, according to the manufacturer’ specifications (Life Technologies). For serum miRNAs analysis, cel-miR-39-specific RT primers and probe (192 and 32 nM, respectively; from Life Technologies) were added to the reverse transcription and pre-amplification reactions, respectively.

### Real time RT-PCR (qPCR)

Expression levels of genes and miRNAs were analyzed in duplicate using gene-specific primers and TaqMan probes (Table S1 in Supplementary Material) and the ABI Prism 7900HT sequence detection system (Life Technologies). Targets raw data (*C*_t_ values) were converted into relative quantities using GeNorm software and then median-normalized and log2-transformed for statistical analysis (11). β-2 microglobulin (β*2M*) and actinβ (*ACT*β) were used as reference genes for target relative quantification. MammaryU6 and RNU48, or miR-16 and miR-39 were used as reference genes for relative quantification of tissue or serum target miRNAs, respectively. The serum of a 3 and 6 months old mouse without grafts was used as control in order to normalize serum miRNAs contents with respect to the animals’ age.

### Statistical analysis

The association between Ki67 levels and PDTXs volume increase was evaluated by Fisher’s exact test. Data were analyzed using Prism 4.0 (GraphPad Inc., La Jolla, CA, USA). The expression profiles of genes and miRNAs in tissue and serum samples were analyzed using the DNA-Chip Analyser Software (http://www.dchip.org) as previously described (12). The variance of miRNAs levels according to xenografts features, such as volume variation, Ki67 score, duration of grafts, and engraftment success was analyzed by ANOVA test provided within DNA-Chip Analyser Software. Differences among samples group were analyzed using the Mann–Whitney *U* test (GraphPad Inc.). Volume variation was considered high (H) when it was >1 and low (L) when it was ≤1. Ki67 levels were considered high (H) when Ki67 immunoreactivity was >50% and low (L) when it was ≤50%.

Statistical significance was assumed if the probability value (*p*) was <0.05.

## Results

### Establishment of PDTXs

**Figure 1:**
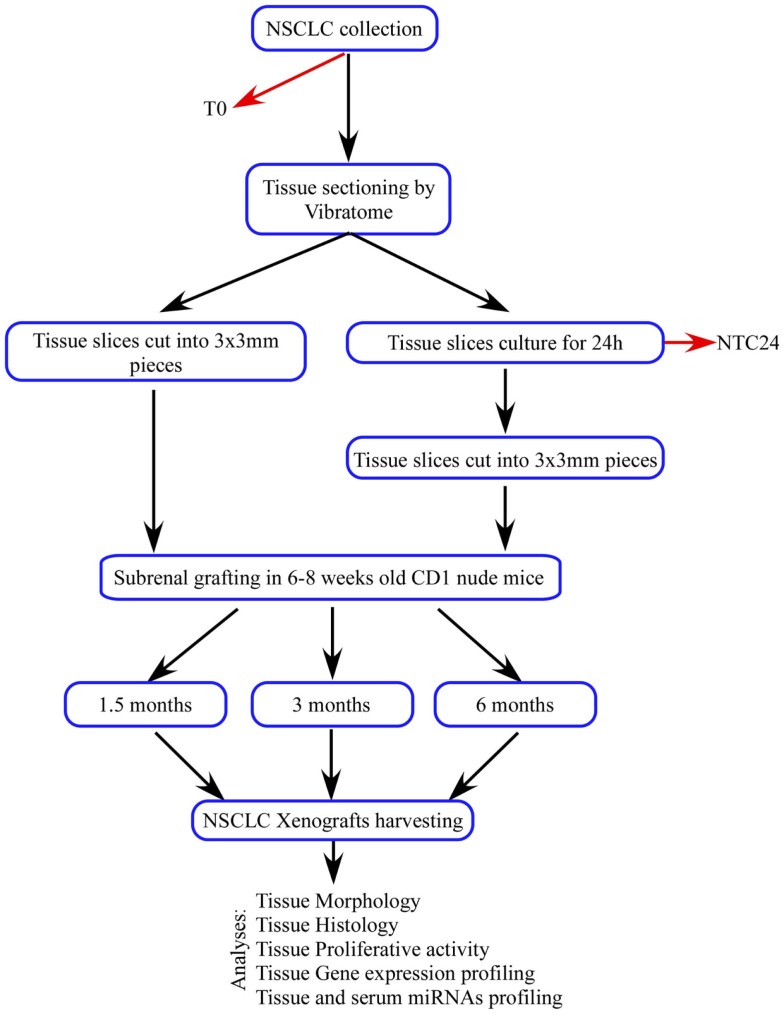
**Schematic overview of the experimental design**.

**Figure 2:**
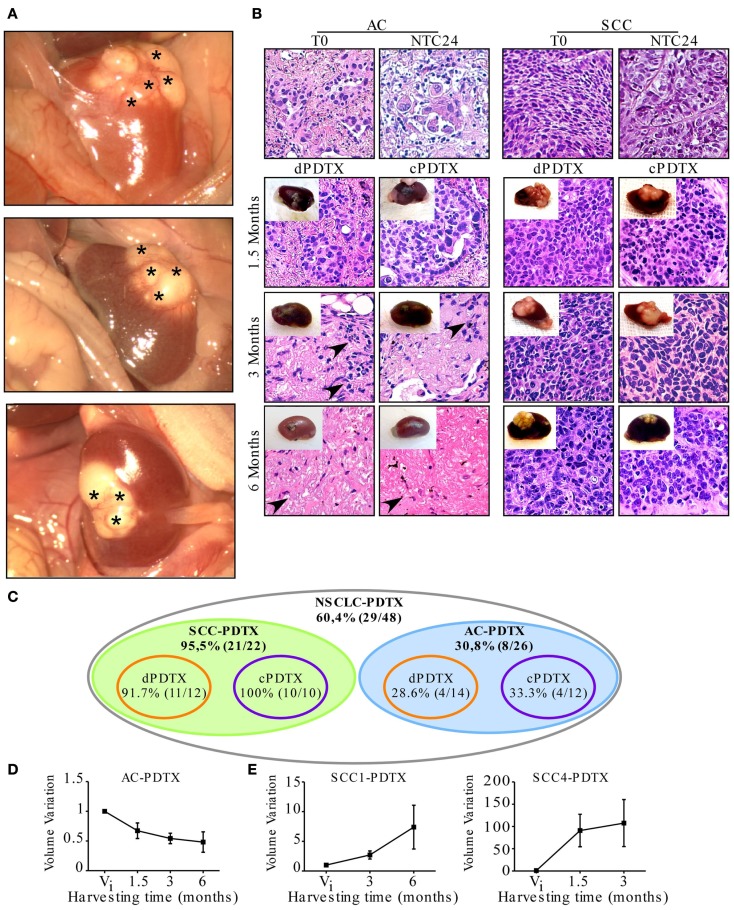
**PDTX establishment from vibratome-generated NSCLC tissue sections**. First generation PDTXs were established by implanting 300 μm-thick NSCLC tissue slices under mice renal capsule. **(A)** Representative macroscopic images of three xenografts at harvesting. Asterisks indicate xenograft superficial vascularization. **(B)** Tissue morphology was determined by H&E and representative images of SCC and AC-PDTXs are shown. AC-PDTXs exhibited the epithelial compartment only at 1.5 months from the graft. The absence of epithelial cancer cells is evident in AC-PTDTXs harvested at 3 and 6 months. Black arrow heads indicate stromal cells. Original magnification is 200×. **(C)** Schematic representation of PDTXs engraftment rates. **(D,E)** Quantification of AC (average of AC2, 3, and 5 samples); **(D)** SCC1 and SCC4-PDTXs. **(E)** Volume variation compared to initial tumor volume (*V*_i_) at different time points, respectively. For all experiments, bars represent mean ± SEM.

Tumor tissue slices of 300 μm thickness were obtained from five patients affected by NSCLC and used to establish 48 first generation PDTXs, as outlined in Figure 1. At mice sacrifice (1.5, 3, and 6 months after implantation) xenografts, and specifically SCC-derived PDTXs displayed evident vascularization (asterisks in Figure 2A). Morphologic analysis of the primary tumors and the corresponding xenografts revealed that 29 PDTXs closely resembled the original cancer (H&E staining; Figure 2B). Conversely, the remaining 19 xenografts (39.6%) did not retain tumor epithelial compartment resulting in a total engraftment rate of 60.4% (Figure 2C). In particular, PDTXs derived from SCC tumors (SCC-PDTXs) showed a higher engraftment rate than xenografts derived from AC tissues (AC-PDTXs, 95.5 vs. 30.8%). AC-PDTXs retained the tumor epithelial component only at 1.5 months from the graft (Figure 2B), whereas these xenografts showed only stromal tissue at 3 and 6 months from the graft as evidenced by microscopic (H&E staining, Figure 2B) and immunohistochemistry (vimentin staining, Figure S1 in Supplementary Material) analyses.

In regards to the *ex vivo* cultures for 24 h of NSCLC tissue slices before grafting, no significant difference in engrafting rate or retention of epithelial compartment was evidenced in cPDTXs compared to dPDTXs. Moreover, dPDTXs and cPDTXs exhibited comparable engraftment rates in SCC (91.7 vs. 100%) or AC (28.6 vs. 33.3%) derived grafts, as shown in Figure 2C.

When we analyzed the xenografts volume variation, we observed that tumors growth was higher in SCC-PDTXs (volume variation range: 0.5–363.8) than AC-PDTXs (volume variation range: 0.17–1.36). Indeed, most of the xenografts derived from AC samples (24/26, 92.3%) did not increase their volume compared to their initial mass (Figure 2D). In contrast, 19 (86.3%) xenografts derived from SCC slices increased their mass compared to their original volume (Figure 2E). Interestingly, SCC-PDTXs showed heterogeneity in tumor growth rate, and two patterns characterized by a slow or fast volume increase were identified. Morphological examination of PDTXs revealed that SCC4-derived xenografts presented large necrotic areas, whereas necrosis was absent in SCC1-PDTXs (not shown). Nevertheless, the difference in cell death did not prevent SCC4-PDTXs to have a higher and faster growth rate than SCC1-derived xenografts (Figure 2E).

Finally, we analyzed liver, lung, contralateral kidney, and spleen for presence of distant metastases at animals’ sacrifice. No metastatic foci were detected by macroscopic or microscopic (H&E) analyses (Figure S2 in Supplementary Material).

### Retention by xenografts of the original cancer biological features

**Figure 3:**
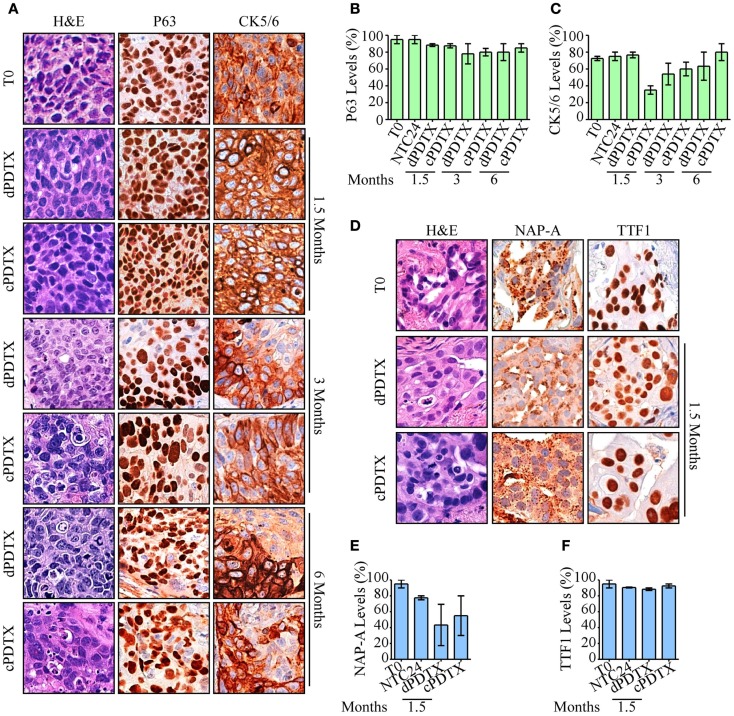
**Morphological and histological analysis of PDTXs**. NSCLC histotypes-specific markers and morphology were analyzed in xenografts. **(A–C)** SCC-derived PDTXs were analyzed for P63 and CK5/6 immunoreactivity at the indicated harvesting schedules and compared to the corresponding human tissue. Representative images of P63 and CK5/6 staining SCC cases are shown in **(A)**, and are quantified in **(B,C)**. Original magnification 200×. **(D–F)** Immunoreactivity of AC-PDTXs and corresponding human tissues for NAP-A and TTF1 markers was analyzed, and a representative AC case is shown. **(D)** Quantification of NAP-A **(E)** and TTF1 **(F)** proteins levels in *T*_0_, NTC24 samples, and relative xenografts at 1.5 months after the graft. Original magnification 200×. For all experiments, bars represent mean ± SEM.

In order to compare primary tumors and corresponding PDTXs, xenografts were investigated for the presence of distinctive histological markers of their original cancer. Therefore, P63 and CK5/6 immunoreactivity was analyzed in xenografts derived from SCC samples (Figures 3A–C), whereas NAP-A and TTF1 stains were investigated in AC-PDTXs (Figures 3D–F). As previously described (8), the levels of distinctive histological markers were preserved after 24 h of *ex vivo* culture (Figure S3 in Supplementary Material).

In xenografts cell type-specific markers, levels were preserved at all harvesting points compared to the original cancer and irrespective of the NSCLC histotype. Moreover, despite a marginal reduction in CK5/6 observed in SCC-cPDTXs at 1.5 months, no significant difference of these proteins levels was observed between dPDTXs and cPDTXs at later harvesting schedules, i.e., 3 or 6 months. TTF1 immunoreactivity was preserved in AC-PDTXs at 1.5 months, whereas NAP-A levels showed a slight reduction in PDTXs tissues that was not statistically significant.

### Proliferation index

**Figure 4:**
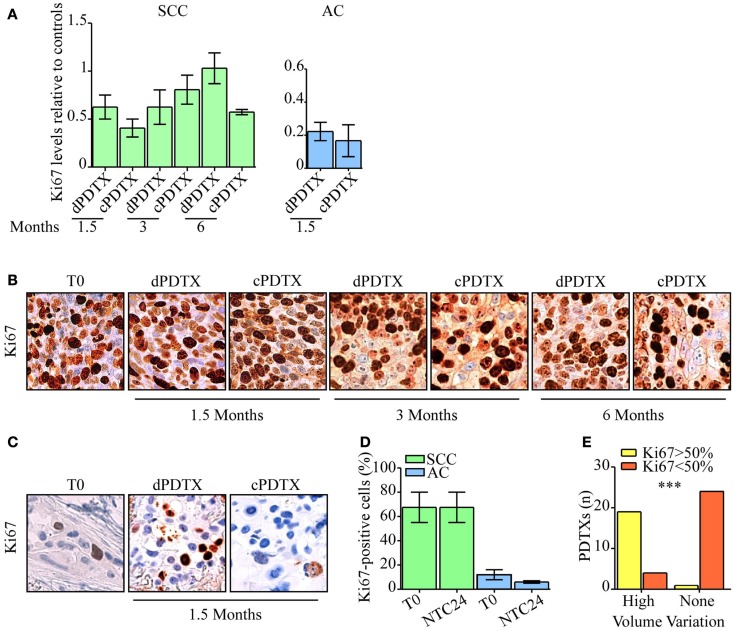
**Evaluation of NSCLC tissues proliferative activity**. Ki67 immunohistochemical levels were analyzed in PDTXs and in their corresponding human cancer tissues. **(A)** Ki67 immunoreactivity is shown for SCC- and AC-PDTXs compared to their relative controls and at the indicated time points. **(B,C)** Representative images of Ki67 staining for SCC- **(B)** or AC5-NSCLC cases **(C)** and matched PDTXs. Original magnification 200×. **(D)** Analysis of Ki67 levels in SCC- or AC-NSCLCs at baseline and after 24 h of *ex vivo* culture. **(E)** The association of high proliferative activity (Ki67 >50%) with PDTXs volume variation was analyzed (high volume variation = volume variation >1). ***p < 0.0001 (Fisher exact test). For all experiments, bars represent mean ± SEM.

Ki67 levels were analyzed in xenografts, in NTC24 cultures, and in the corresponding human cancer. No loss of tissue proliferative potential was evidenced between baseline (*T*_0_) and NTC24 samples. Regarding xenografts, SCC-PDTXs maintained high Ki67 levels compared to their primitive cancer and NTC24 at all time points (Figures 4A,B). No significant difference in Ki67 immunoreactivity was shown between dPDTX and cPDTX derived from SCC samples. Conversely, AC-PDTXs exhibited lower Ki67 levels than their original cancer (Figures 4A,C). Nevertheless, no significant difference was highlighted at 1.5 months after the graft between dPDTX and cPDTX derived from AC samples.

We therefore looked for differences in proliferative potential in human NSCLC samples that could account for the distinct behavior of SCC-PDTXs respect to AC-PDTXs. SCC tissues showed higher proliferative index than AC samples at baseline (Figure S4 in Supplementary Material) or after 24 h of *ex vivo* culture (Ki67 score range: 55–80 or 5–20%, respectively; Figure 4D). In particular, SCC4 tumor showed higher Ki67 levels than SCC1 (80 vs. 55% of Ki67-positive cells; Figure S4 in Supplementary Material). Moreover, PDTXs generated from tissues exhibiting an elevated proliferative index (Ki67-positive cells >50%) significantly displayed a higher growth *in vivo* (volume variation >1, *p* < 0.0001; Figure 4E).

### Expression analysis of genes related to a stem cell phenotype

**Figure 5:**
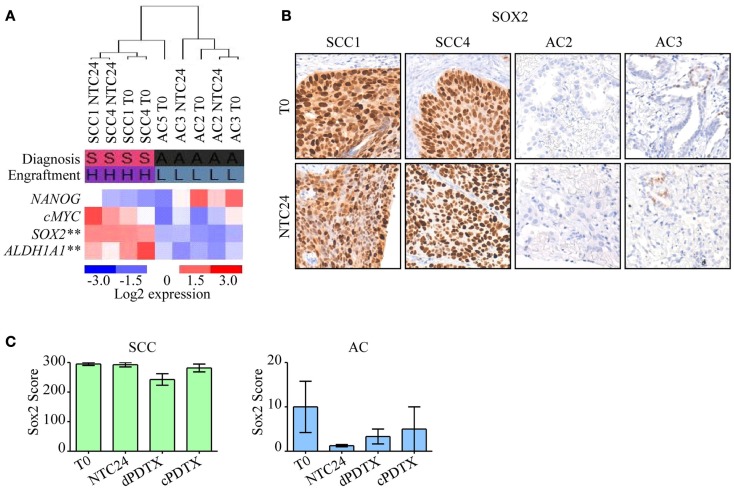
**Analysis of stem cell-related genes in PDTXs and corresponding human tumors**. Expression profiles of stem cell-related genes were investigated in SCC or AC samples at baseline. **(A)** Heatmap of *NANOG*, *c-MYC*, *SOX2*, and *ALDH1A1* expression in NSCLC tissues at *T*_0_ and after 24 h of *ex vivo* culture. *SOX2* and *ALDH1A1* were significantly more expressed in SCC cases, and in PDTXs with successful engraftment (**p < 0.001, by ANOVA). Diagnosis: S, SCC; A, AC. Engraftment: H, high; L, low. **(B)** Representative SCC and AC cases stained with an antibody directed against SOX2 at *T*_0_ and after 24 h of *ex vivo* cultures (NTC24). Original magnification 200×. **(C)** SOX2 immunoreactivity is shown for SCC- and AC-PDTXs and their relative controls. For all experiments, bars represent mean ± SEM.

To gain insights on the different SCC and AC engraftment rates, the expression levels of four stem cell-related genes, namely *ALDH1A1*, *c-MYC*, *NANOG*, and *SOX2* (Table S1 in Supplementary Material), were analyzed in *T*_0_ and in NTC24 samples. The gene expression analysis identified *SOX2* and *ALDH1A1* as the most deregulated transcripts in SCC compared to AC samples at baseline and after 24 h of *ex vivo* culture, with an increased expression of more than 100- or 16-folds in SCC tumors respect to AC. Moreover, *SOX2* and *ALDH1A1* expression levels were significantly higher in SCC tissues compared to AC and correlated with the engraftment success (*p* < 0.001 by ANOVA; Figure 5A). *c-MYC* was also more expressed in SCC than in AC tissues (7- and 36.4-fold differences for *T*_0_ and NTC24 samples, respectively), whereas *NANOG* showed a mild overexpression in AC-NSCLC tissues at baseline (0.5-fold difference) that was not confirmed in NTC24 samples (1.6-fold difference). Finally, *c-MYC* and *NANOG* expression levels were not correlated to the engraftment rates.

SOX2 protein levels were investigated by immunohistochemistry in control tissues (*T*_0_ and NTC24 samples), and in their corresponding xenografts. Confirming gene expression data, SOX2 protein levels were more elevated in SCC than AC samples (Figure 5B). SOX2 expression was maintained after 24 h of *ex vivo* culture and in xenografts derived from SCC tissue slices (Figure 5C). In contrast, SOX2 was poorly expressed by AC samples, either in *T*_0_, NTC24 samples or in their corresponding PDTXs at all harvesting points (Figures 5B,C). No significant differences were demonstrated in SOX2 staining between dPDTX and cPDTX derived from either SCC or AC lung cancers (Figure 5C).

### Tissue and serum miRNAs expression analysis

Lastly, to investigate whether our model supported the discovery of new biomarkers, we analyzed in SCC tissues or serum the expression of seven miRNAs (miR-19a, miR-19b, miR-20a, miR-21, miR-31, miR-150, and miR-210) known to be involved in lung cancer (13, 14).

**Figure 6:**
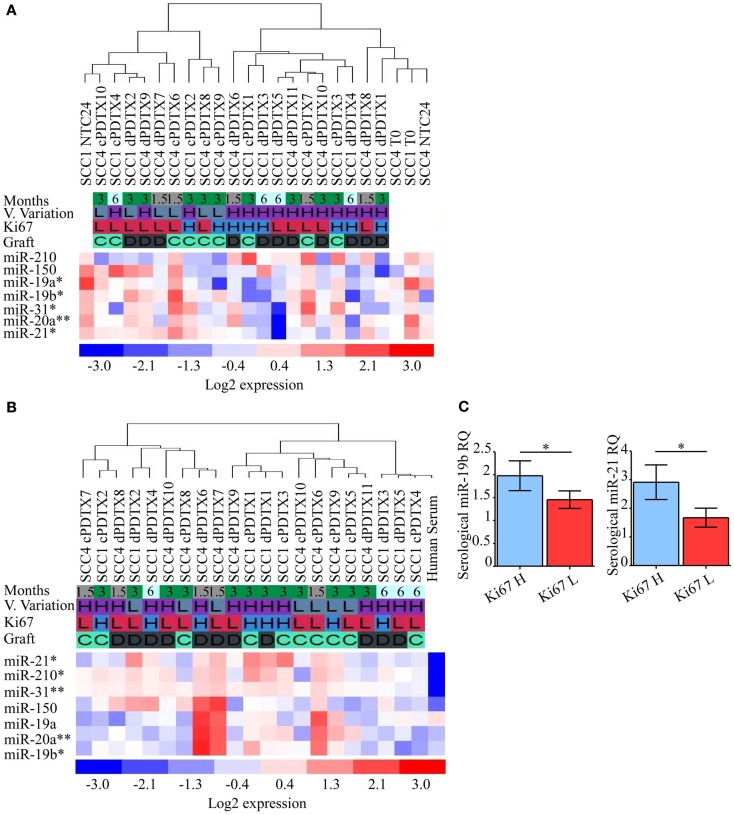
**Analysis of NSCLC-related miRNAs in PDTXs, corresponding human tumors, and serum**. Expression levels of miRNAs involved in lung cancer progression were analyzed in PDTXs or human tumor tissues and serum derived from SCC patients. **(A)** Heatmap of SCC1, SCC4, and their relative PDTXs tissues analyzed for miRNAs expression levels. miR-19a levels were inversely correlated with Ki67 immunoreactivity (*p = 0.03 by ANOVA). Tissue levels of miR-19b, -20a, -21, and -31 significantly decreased at 6 months compared to the PDTXs harvested at 1.5 and 3 months (respectively *p = 0.05, **p = 0.004, *p = 0.01, and *p = 0.03 by ANOVA). **(B)** Heatmap of SCC1- and SCC4-PDTXs serum analyzed for miRNAs expression levels. miR-19b, miR-20a, and miR-31 were significantly higher in 1.5 months old PDTXs (respectively *p = 0.02, **p = 0.007, and **p = 0.003, by ANOVA), and miR-21 and -210 levels were correlated with PDTXs proliferative activity (respectively *p = 0.03, *p = 0.02 by ANOVA). **(C)** PDTXs with high Ki67 levels (>50%) exhibited significantly high circulating miR-19b and -21 levels (*p = 0.04 and *p = 0.03 by Mann–Whitney *U* test, respectively). For all experiments, bars represent mean ± SEM. V. Variation, volume variation; H, high; L, low; C, graft after 24 h culture; D, direct graft.

Variation of miRNAs expression levels in SCC-PDTXs tissues (Figure 6A) or serum (Figure 6B) was investigated relative to xenografts proliferation, volume variation, and type and duration of the graft. MiR-19a levels in PDTX tissues were inversely correlated with Ki67 immunoreactivity (*p* = 0.03 by ANOVA; Figure 6A). Whereas tissue levels of miR-19b, -20a, -21, and -31 significantly decreased at 6 months compared to the earlier PDTX harvesting points (*p* = 0.05, *p* = 0.004, *p* = 0.01, and *p* = 0.03 by ANOVA, respectively; Figure 6A). Globally, tissue miRNAs expression levels showed a general downregulation in PDTXs compared to baseline *T*_0_ and NTC24 samples.

Serological analysis of the aforementioned miRNAs documented that circulating miR-21 and miR-210 levels were differentially modulated according to PDTXs proliferative activity (*p* = 0.03, *p* = 0.02 by ANOVA, respectively; Figure 6B). Conversely, serological levels of miR-19b, miR-20a, and miR-31 significantly decreased at the latter PDTX harvesting point compared to the 1.5 and 3 months schedule (*p* = 0.02, *p* = 0.007, and *p* = 0.003 by ANOVA, respectively; Figure 6B), mimicking what observed for these small regulatory RNAs in PDTXs tissues (Figure 6A).

In particular, PDTXs with a Ki67 immunoreactivity >50% showed significantly higher expression of circulating miR-19b and miR-21 levels (*p* = 0.04 and *p* = 0.03 by Mann–Whitney *U* test, respectively; Figure 6C).

No other correlations could be evidenced between miRNAs and PDTX features.

## Discussion

The improvement of *in vivo* models that closely mimic primary NSCLC and that recapitulate tumor heterogeneity at molecular and morphological level is a crucial element to pursue a personalized therapeutic approach and to discover new targets for innovative treatments. In this context, the PDTXs experimental system represents a noteworthy opportunity. Furthermore, *in vivo* models of human tumors could provide novel prognostic biomarkers besides contributing to innovative therapeutic targets discovery and testing.

To address these ambitious goals, technical procedures must be optimized and standardized to ensure feasibility and reproducibility of PDTX generation from small tumors, the majority of currently resected NSCLCs. In addition, the possibility to use samples from primary tumors in a deferred setting, and not immediately after surgery, could strongly expand the usefulness of tumor xenografts.

In this scenario, our study implemented the PDTX model by implanting 300 μm-thick, precision-cut NSCLC tissue slices under the renal capsule of CD1 athymic mice and by *ex vivo* culturing procedures of tumor sections before grafting. PDTX models are entering the research laboratories to study tumor biology (10, 15, 16) and anticancer therapy (4, 17), for different malignancies. To date, scarce data are available regarding the establishment of PDTX from precision-cut tissue slices (18, 19) and the majority of previously reported NSCLC xenograft models were established from minced tissues (10, 20–22). Our model has the potential to provide several improvements compared to PDTX derived from minced tumor tissues. Reduced tissue thickness might improve the proper exchange of nutrients and drugs. Furthermore, tissue slices obtained with vibratome sectioning reduces tissue injury compared to mincing and allows the achievement of implantable tissues with accurate and uniform dimensions (18). With the precision-cut technique presented here, more tumor sections from the same samples can be obtained, compared to the tissue mincing technique. Lastly, tissue sectioning by vibratome ensures a much greater reproducibility of the implanted xenografts in different animals and allows greater consistency of the tumor cell population present in the serial sections.

The possibility to culture *ex vivo* tumor sections for 24 h before engrafting allows to delay graft without causing tissues damage, loss of tumor viability, or alterations of morphological features.

Patients-derived tumor xenograft models show three principal applications. First, they are useful to test new anticancer drugs in a context that considers both a patient-derived tumor tissue and the *in vivo* situation. Second, PDTX method is appropriate to identify biomarkers of response to therapies and, third, it could support the delineation of a personalized therapeutic algorithm. Indeed PDTXs may allow the preclinical evaluation of patients’ tumor sensitivity to specific therapies *in vivo*.

One limitation of the previously described techniques is the use of minced tumor tissues or of tumor pieces. These procedures require a larger amount of tumors than precise-cut tissue slices and, moreover, they did not grant a controlled or homogeneous tissue grafting among the different receiving mice per human tumor. The availability of large tumors is expected to decrease due to early prevention and the use of neo-adjuvant regimens before surgery. Furthermore, this topic is particularly relevant in lung cancer since advanced disease is not surgically treatable.

Our study improves the use of PDTXs models because it allows the generation of serial xenografts starting from small tumor masses. The establishment of PDTXs from small tumors is useful for clinical applications where tissues availability is limited. Moreover, PDTX obtained from thin organ slices are more homogeneous and this may facilitate their implantation compared to minced specimens.

Lastly, the possibility to culture tumor sections for 24 h grants a time-frame that can be exploited to modulate important signaling pathways or to delay grafting to a more convenient moment.

The data obtained from the established 48 NSCLC-PDTXs show that PDTX tumors reproduce the histological features and the histotype distinctive markers of patients’ primary cancers up to 6 months after the graft. Moreover, tissue culture does not affect PDTX ability to preserve original tumors’ characteristics nor does it affect engrafting rate, PDTX growth, or xenografts’ proliferative potential.

Pertaining the implantation site of PDTXs under the renal capsule, our data confirm that this is suitable to obtain vascularization of the xenograft. As described in the literature, the renal capsule improves tumor engraftment due to the development of rich vascularity, which is a key feature for graft survival especially during the initial phase after implantation (16). The use of thin tumor tissue slices has improved this procedure, and the presence of vascularization was noted initially in all SCC- and AC-PDTXs, irrespective of the final engraftment success.

However, our data suggest that host-mediated vascularization of the PDTX is not sufficient for graft survival and expansion. In fact our results show that PDTX growth is more dependent on the proliferative ability of the primary cancer. Moreover, other core elements such as stem-related factors may be involved in adaptation of the xenograft in the host microenvironment, determining a successful engraftment.

We also show that the tumor histotype is importantly involved in PDTX survival and expansion. As previously reported in literature in different settings (23, 24), our model highlighted that SCC samples show a higher engraftment ability than AC specimens. Indeed, SCC samples that showed elevated Ki67 levels generated SCC-PDTXs that retained a high proliferative index and increased tumor volume.

Albeit limited by the small number of primary tumors used in this study, no correlation was observed between the engraftment rate and other clinical features, such as lymph node status, tumor grade, and size. These data indicate that tumor ability to engraft and survive is more often associated with elevated proliferative potential rather than aggressive features such as presence of nodal metastases. Future studies with larger clinical series are needed to confirm these conclusions.

However, to date, the main determinants affecting PDTX success rate are still unknown. In order to understand other factors involved in tumor engraftment, we analyzed the expression levels of four stem cell-related genes at baseline levels in this set of human NSCLCs. *SOX2* and *ALDH1A1* expression were more elevated in SCC specimens than in AC tissues. In particular, *SOX2* levels were retained by SCC-PDTXs and lost by AC-PDTXs compared to their controls. *SOX2* is a stem cell transcription factor that plays a crucial role in the regulation of embryonic development and it is one of the genes involved in reprograming human somatic cells to pluripotent stem cells (25). Overexpression of *SOX2* has been described in lung cancer, in particular in SCC, where it is frequently amplified and promotes cancer progression (26). Our data show that this stem-factor is overexpressed at either the gene or protein level in SCC-PDTXs, therefore suggesting that *SOX2* favors tumor cell survival and adaptation to different microenvironments, eventually sustaining tumor growth.

Lastly, in order to investigate the suitability of our model for novel molecular biomarkers discovery, we analyzed tissue and serum miRNAs expression in PDTXs and corresponding human SCC tissue. As a proof of concept, we analyzed miRNAs involved in lung cancer.

Human miRNAs were preserved in murine hosts and could be detected in serum and tissue of our PDTXs model. In particular, circulating miR-19b, -21, and miR-210 levels were directly correlated with PDTXs proliferation. As a member of the miR-17-92 cluster, miR-19b is an oncogenic key factor present in different types of cancer (27, 28). Recently, miR-19b has been shown to induce tumor growth and metastasis *in vivo* (29). Moreover, circulating miR-19b levels have been associated with a worse disease progression in patients affected by NSCLC (30). miR-21 acts as an oncogenic miRNA and is involved in the regulation of cell cycle, apoptosis, invasion, and metastasis. Recent studies demonstrated that miR-21 promoted cell proliferation (31, 32) and its inhibition induced a cell cycle arrest at G2/M phase (31). MiR-210 is a hypoxia-inducible miRNA in lung cancer and melanoma (33) and it has been detected in exosome *in vitro* and *in vivo* (34). Moreover, serological miR-210 levels were significantly upregulated in NSCLC patients compared to healthy controls and were correlated with an advanced disease (35). In line with the literature, we found increased circulating miR-210 levels in PDTX characterized by elevated tumor proliferation.

These results suggest that our model could be a useful tool for biomarker discovery, allowing time-course investigations over several months of tumor engraftment and growth.

Overall, our study indicates that PDTXs established from precise-thick tissue slices represent a valid tool to investigate cancer biology and to discover novel biomarkers. Our results highlight the importance of PDTX models to achieve a personalized therapeutic approach with *in vivo* analysis and the monitoring of tumors response to anticancer agents.

## Author Contributions

Author contributions included: work concept and design (MR, AF, SG, VV, SB), data acquisition (MR, AF, DR, AG, AP, SF), analysis (MR, AF, SG, ADG, SF, AP), and interpretation (MR, AF, SG, AG, SF, VV, SB), drafting of the manuscript (MR, AF, SG, VV), editing of the manuscript (AF, VV, SB), approval of final content for journal submission and publication (MR, AF, SG, AG, SF, DR, AP, VV, and SB).

## Conflict of Interest Statement

The authors declare that the research was conducted in the absence of any commercial or financial relationships that could be construed as a potential conflict of interest.

## Supplementary Material

The Supplementary Material for this article can be found online at http://www.frontiersin.org/Journal/10.3389/fonc.2015.00052/abstract

## Abbreviations

AC, adenocarcinoma; DAB, 3,3′-diaminobenzidine; FFPE, formalin-fixed and paraffin-embedded; H&E, hematoxylin and eosin; NSCLC, non-small cell lung cancer; NTC24, 24 h culture control; PDTXs, patients-derived tumor xenografts; cPDTXs, patients-derived tumor xenografts derived from cultured tissues; dPDTXs, patients-derived tumor xenografts derived from fresh tissues; RQ, relative quantity; SCC, squamous cell carcinoma; *T*_0_, baseline time; *V*_f_, final volume; *V*_i_, initial volume.
