# Supplementary material for: A New Mouse Avatar Model of Non-Small Cell Lung Cancer
Source: Front Oncol. 2015 Mar 3;5:52. doi: 10.3389/fonc.2015.00052 (PMC4347595; doi:10.3389/fonc.2015.00052)

**MS. 127746_REV2**

**A new Mouse Avatar model of Non Small Cell Lung Cancer**

Maria Veronica Russo, Alice Faversani, Stefano Gatti, Dario Ricca, Alessandro Del Gobbo, Stefano Ferrero, Alessandro Palleschi, Valentina Vaira and Silvano Bosari

**Supplementary Material**

Supplementary Table S1

Supplementary Figures S1-4 and corresponding Legends

Table S1. **Stem cell-related genes analyzed in NSCLC tissues and PDTXs.** Primers and probes (Assay ID, Life Technologies) used for the study, and a brief description of the genes function (adapted from GeneCards V3 Human Genes Database; http://www.genecards.org/) are provided.

| **Gene Symbol** | **Gene Name** | **Gene description and function** | **Assay ID** |
| --- | --- | --- | --- |
| *ALDH1A1* | Aldehyde Dehydrogenase 1 Family, Member A1 | Aldehyde dehydrogenase is the next enzyme after alcohol dehydrogenase in the major pathway of alcohol metabolism.  This gene encodes the cytosolic isozyme. | Hs00946916_m1 |
| *cMYC* | V-Myc Avian Myelocytomatosis Viral Oncogene Homolog | The protein encoded by this gene is a multifunctional, nuclear phosphoprotein that plays a role in cell cycle progression, apoptosis and cellular transformation. It functions as a transcription factor that regulates  transcription of specific target genes. | Hs00153408_m1 |
| *NANOG* | **Nanog** Homeobox | Transcription regulator involved in inner cell mass and embryonic stem (ES) cells proliferation and  self-renewal. Imposes pluripotency on ES cells and prevents their differentiation towards extraembryonic endoderm and trophectoderm lineages. | Hs04260366_g1 |
| *SOX2* | SRY(Sex Determining Region Y)-Box 2 | This intronless gene encodes a member of the SRY-related HMG-box (SOX) family of transcription factors involved in the regulation of embryonic development and in the determination of cell fate. The product of this gene is required for stem-cell maintenance in the central nervous system, and also regulates gene expression in the  stomach. | Hs01053049_s1 |
| *ACTβ* | *β*-Actin | Housekeeping gene | Hs99999903_m1 |
| *β_2_M* | *β*2-Microglobulin | Housekeeping gene | Hs99999907_m1 |
| cel-miR-39 | cel-miR-39 | Endogenous Control | 000200 |
| hsa-miR-16 | hsa-miR-16 | Endogenous Control | 000391 |
| RNU48 | RNU48 | Endogenous Control | 001006 |
| U6 snRNA | U6 snRNA | Endogenous Control | 001973 |
| hsa-miR-19a | hsa-miR-19a | microRNA | 000395 |
| hsa-miR-19b | hsa-miR-19b | microRNA | 000396 |
| hsa-miR-21 | hsa-miR-21 | microRNA | 000397 |
| hsa-miR-150 | hsa-miR-150 | microRNA | 000473 |
| hsa-miR-210 | hsa-miR-210 | microRNA | 000512 |
| hsa-miR-20a | hsa-miR-20a | microRNA | 000580 |
| hsa-miR-31 | hsa-miR-31 | microRNA | 001100 |

**Supplementary Figures**

Figure S1. **AC-PDTXs Vimentin staining.** A representative image of an AC-PDTX stained with H&E and with an antibody against Vimentin is shown. Original Magnification x1000.


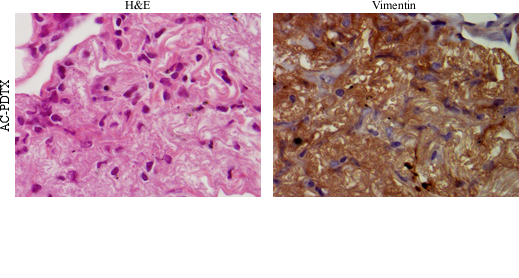


Figure S2. **Evaluation of metastatic foci in PDTXs.** Representative H&E images of controlateral kidney, liver, lungs and spleen collected at mice sacrifice from a SCC-PDTX, a AC-PDTX or a control animal. Original Magnification x100.


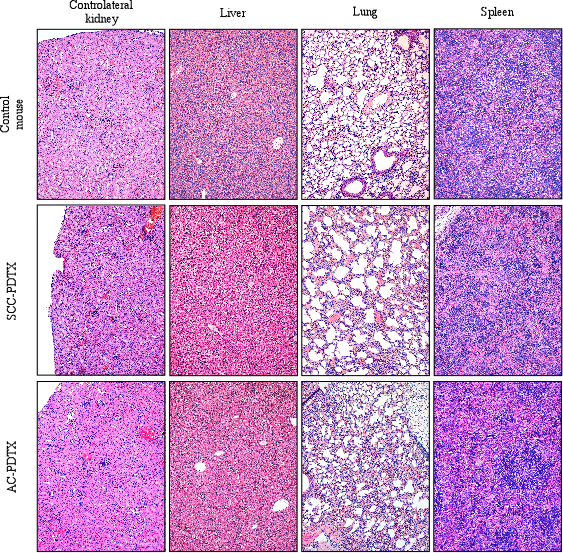


Figure S3. **NSCLC tissues after 24 h of *ex vivo* culture preserve morphology and histotype-specific markers.** Histological markers specific for squamous cell carcinoma (SCC, A) or adenocarcinoma (AC, B) NSCLC histotype were analyzed at baseline (T0) and in the corresponding organotypic tissue slice after 24 hours of *ex vivo* culture (NTC24). Tumor morphology or proliferative activity was assessed by hematoxylin and eosin (H&E) or Ki67 staining, respectively. P63, tumor protein p63; CK5/6, Cytokeratin 5/6; NAP-A, Napsin A; TTF1, transcription termination factor. Original Magnification x200.


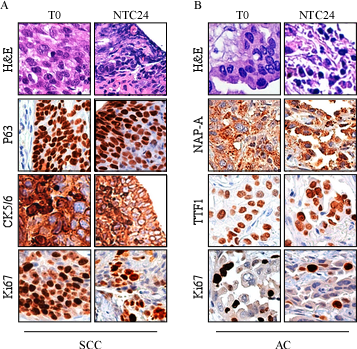


Figure S4. **Basal** **proliferative activity of SCC and AC tumors.** Representative images of human NSCLC samples used for PDTX generation. Tumor morphology (H&E) and proliferative activity (Ki67 immunohistochemistry) are shown. Original Magnification x50.


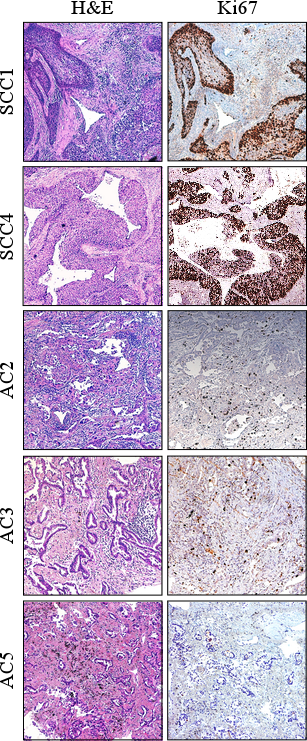

Supplement: Supplementary file 1 [file data_sheet_1.docx]
